# Negative Life Events and Procrastination among Adolescents: The Roles of Negative Emotions and Rumination, as Well as the Potential Gender Differences

**DOI:** 10.3390/bs13020176

**Published:** 2023-02-15

**Authors:** Lizhong Liu, Tianyi Zhang, Xiaochun Xie

**Affiliations:** 1 School of Education, Central China Normal University, Wuhan 430079, China; 2 School of Psychology, Northeast Normal University, Changchun 130024, China

**Keywords:** negative life events, negative emotions, rumination, procrastination, moderated mediation model, gender differences

## Abstract

Procrastination (the intentional delay of action despite knowing that one will be worse off due to the delay) is a widespread phenomenon with various negative consequences, especially among adolescents. Based on relevant evidence, this study examined the relation between negative life events and adolescents’ procrastination, as well as the underlying mechanisms—specifically, the effects of negative emotions and rumination, as well as the potential gender differences. A total of 780 adolescents (M_age_ = 12.92 years old; 52.2% females) were recruited to complete a set of questionnaires assessing negative life events, procrastination, depression-anxiety-stress symptoms and rumination. Results showed that negative life events were positively associated with procrastination, and negative emotions significantly mediated the relation; rumination played a moderating role in this mediation model, specifically, both the direct and indirect effects in this mediation model were stronger for adolescents with higher rumination. Besides this, gender differences in this moderated mediation model were also found—the indirect effect of negative emotions was stronger for girls, and this mediating effect could be moderated by rumination only for boys. These results expanded our understanding of how negative life events influence procrastination and when (or for whom) negative life events influence procrastination the most. The findings also have significant implications for the prevention and intervention of adolescents’ procrastination.

## 1. Introduction

As a common and widespread phenomenon, procrastination refers to the intentional delay of action despite knowing that one will be worse off due to the delay [1,2]. For example, even if some junior students know that they must do a good job of preparation before the exam, they will still put off the preparation work until it cannot be put off [3]. Procrastination has been proved to be irrational predominantly and is defined as maladaptive behavior, due to the fact that it usually leads to irrational delay due to self-regulatory failure [4]. It can take a heavy toll on individuals’ life satisfaction and mental health [5,6]. For instance, Aftab et al. [4] found that procrastination was positively associated with depression. Therefore, previous studies have also investigated the influencing factors of procrastination. Previous studies showed that negative affect and inhibition [2], high impulsivity [7] and lack of self-control [4] were all associated with individuals’ procrastination. Our study focused on exploring the relationship between negative life events and adolescents’ procrastination and revealing the psychological mechanisms and conditions underlying this relationship. 

Previous research has examined the influencing factors of procrastination from various perspectives, such as parenting patterns [8], negative emotions [9], attention control [10], self-worth [8], maladaptive behavior [1] and personality traits [11,12]. Although previous studies have revealed the influencing factors of procrastination from the different perspectives mentioned above, few studies have explored the effects of the combination of these factors on procrastination. Given that procrastination is associated with various factors, procrastination may also be associated with a combination of factors. Therefore, to further understand the causes of individuals’ procrastination, researchers should consider environmental factors (e.g., negative life events), individual emotional factors (e.g., negative emotions) and personality factors (e.g., rumination) in a relatively comprehensive model.

### 1.1. Negative Life Events and Procrastination

Negative life events refer to unpleasant events that cause individuals to experience emotional problems and develop their negative emotions [13]. Adolescence is a critical period when individuals can be overwhelmed with negative life events, such as academic performance (e.g., failure in examinations, excessive study burden), interpersonal communication (e.g., being misunderstood and misperceived by others and disputes with classmates or friends), and family and social environment (e.g., conflicts within their families and their important items being stolen or lost) [14]. Negative life events were associated with adolescents’ subsequent internalizing and externalizing problems [15]. Previous studies have found that adolescents experiencing stressful life events may experience psychological distress, including depression, anxiety and stress, and even develop maladaptive behaviors such as suicidal behaviors [13,16,17]. Few studies, however, have explored the direct relationship between negative life events and procrastination in adolescence and the mechanisms underlying this link. Both negative life events and procrastination are important parts of adolescents’ daily life. Some indirect evidence also suggests that negative life events may affect procrastination in adolescence. 

First, negative life events can exert a serious negative impact on individuals’ self-concept, resulting in low self-esteem [18]. Given that avoiding failure or negative consequences is the first thing individuals with low self-esteem care about, they tend to engage in procrastination-relevant behaviors to avoid aversive or difficult tasks [19]. Moreover, low self-esteem means low self-confidence and high self-handicaps [20]. Empirical research has also demonstrated that self-handicapping was associated with procrastination [21]. Therefore, adolescents suffering from negative life events tend to have low levels of self-esteem and develop procrastination-relevant behaviors. Second, individuals suffering from negative life events may lose their self-control resources and develop poor self-control, which may trigger procrastination. Li, Zhang, Li, Zhen and Wang [22] showed that stressful life events were associated with poor effortful control. Given that procrastination essentially reflects self-regulatory failure resulting from low levels of effortful control [3], adolescents exposed to negative life events may engage in procrastination-relevant behaviors. Empirical research also demonstrated that self-handicapping was associated with procrastination [21]. Therefore, negative life events may cause adolescents to procrastinate on aversive or difficult tasks by reducing their self-esteem and increasing their self-handicapping. Based on the above argument, this study will examine the relationship between negative life events and procrastination in adolescence and suppose that adolescents suffering from negative life events may have more procrastination (Hypothesis 1a). 

Although negative life events have been linked to more procrastination, the psychological mechanisms and conditions underlying this link remain unclear. Due to the absence of the intrinsic mechanisms and conditions connecting negative life events to procrastination, only limited practical guidance can be offered for adolescents as well as for educators and parents to develop intervention strategies. The individual–context interaction theory also points out that behavioral problems may be the result of the interaction of environmental factors and individuals’ maladaptive personality traits [23]. Therefore, to fill this gap and refine our understanding of the link between negative life events and adolescents’ procrastination, the present study will include the intervening and contextual factors in a combination model (moderated mediation model) to reveal how and when negative life events link to procrastination. Specifically, this study will take negative emotions as a mediator and rumination as a moderator to reveal the mechanisms and conditions in the link between negative life events and procrastination.

Besides this, gender differences in negative emotions and rumination have been observed among adolescents. Girls tend to ruminate more in early adolescence and experience more negative emotions [24]. The link between potential antecedent variables and procrastination may also show gender differences [8]. For instance, Zhou [25] demonstrated that personality traits play different roles in active and passive procrastination in different gender groups. There may also be gender differences in the mechanisms and conditions of the link between negative life events and procrastination. Therefore, gender will also be taken into account as a contextual factor in this study to reveal the gender differences of the mechanisms and conditions in the link between negative life events and procrastination. 

Moreover, previous studies on the relationship among negative life events, negative emotions, rumination and procrastination are mostly based on the Western cultural context [3,26]. Considering the potential differences between the Eastern and Western cultural context, this study will take adolescents in the Eastern cultural context as subjects to reveal the mechanisms and conditions in the link between negative life events and procrastination.

### 1.2. Negative Emotions as a Mediator

Emotion typically refers to the individual’s subjective experience of the social and physical world [27]. Generally, emotion can be divided into positive emotions and negative emotions. Negative emotions typically refer to individuals’ subjective feelings about their unsatisfactory needs, failures, and other negative life events. It is mainly composed of symptoms of depression, anxiety and stress [28,29]. The positive correlation between negative life events and negative emotions has been proved by many previous studies. Franko et al. [30] demonstrated that stressful life events or stressors were positively associated with depressive symptoms in the next few years. An empirical study also showed that students suffering from more stressful life events may experience more depressive symptoms [31]. Negative life events were also positively associated with anxiety [32]. Moreover, negative life events showed a positive association with stress [33,34]. Above all, adolescents suffering from more negative life events may perceive more negative emotions. 

Emotion plays a decisive role in humans’ behavior and decision-making processes [35]. Positive emotions can promote individual adaptive behavior, such as goal-seeking behaviors and prosocial and altruistic behaviors [36], whereas negative emotions may result in maladaptive behaviors, such as antisocial behavior [37]. Negative emotions have also been recognized as an important factor that may affect procrastination. According to construal level theory (CLT), negative emotions cause people to seek full enjoy immediately and neglect the pursuit of long-term goals or higher achievements [38]. Thus, individuals experiencing negative emotions may give up completing difficult tasks or put off tasks as long as possible. The broaden-and-build theory posits that individuals may have poor self-esteem, self-confidence and self-efficacy when sinking into negative emotions [39]. Individuals who experience negative emotions may doubt their ability to complete the tasks successfully and believe that any failure means their incompetence [3]. Besides this, procrastination, as quintessential self-regulatory failure, can be considered a dysfunctional strategy of mood-regulation [26]. Individuals who have experienced negative emotions may repair their bad mood by avoiding aversive tasks over a short period of time, which in turn develops into procrastination. Empirical research also shows that participants who underwent a negative mood induction spent more time procrastinating and not preparing for the next task in the study [9]. Therefore, individuals with negative emotions are more likely to engage in procrastination. Empirical research also reveals a positive correlation between negative emotions and procrastination [40,41]. Given that negative emotions were associated with negative life events [31] and negative emotions showed a positive correlation with procrastination, negative emotions may play a mediating role in the relation between negative life events and procrastination. (Hypothesis 1b).

### 1.3. Rumination as a Moderator

Negative life events were believed to be associated with the increases in negative emotions and procrastination. However, not all adolescents suffering from negative life events have the same levels of negative emotions and procrastination. In other words, some individual variables (e.g., rumination) may act in a catalytic role in the link between negative life events and negative outcomes. Therefore, rumination was tested as a moderator in the present study. Specifically, we explored whether rumination could moderate the links between negative life events and negative emotions and procrastination. 

Rumination was originally used to describe the habit of regurgitating food and chewing it again in animals. With the development of psychology, rumination was used to describe the phenomenon in which humans repetitively and passively center on the causes and potential adverse outcomes of the negative life events, instead of taking measures to cope with or solve the problems they face [42]. Rumination is believed to be a relatively stable personality trait that acts as a catalytic in the process by which negative life events induce internalizing and externalizing problems [13,43]. That is, individuals who tend to ruminate to a higher extent will experience more emotional or behavioral problems than those who do not. The response styles theory indicates that individuals with high levels of rumination are accustomed to repetitively and passively centering on the causes and potential adverse consequences of negative life experiences rather than taking constructive action to cope with these events [42]. By contrast, individuals with low levels of rumination prefer to face negative life events rationally and cope with the problems gradually. Therefore, individuals with high levels of rumination may have more emotional and behavioral problems when suffering from negative life events. 

Empirical studies have demonstrated that rumination could moderate the effects of negative life events on internalizing or externalizing problems. For instance, Kraaij et al. [13] showed that the effect of negative life events on depressive symptoms was stronger for those who used ruminative style to a higher extent than those who did not. Although empirical research has not revealed a moderating effect of rumination on the link between negative life events and procrastination, rumination has shown positive and significant correlation with procrastination [44]. Previous studies have also indicated that individuals with higher rumination tend to have lower self-control and self-regulation [45]. Procrastination has been considered a self-regulation deficit manifesting itself in problems of self-regulation [3]. Therefore, it is reasonable to assume that adolescents with higher rumination may have more procrastination when suffering from negative life events.

More important, rumination could act as a moderator in the mediation model consisting of negative life events, negative emotions and procrastination. Rumination will moderate the effect of negative life events on procrastination, with this effect being stronger for adolescents with a higher level of rumination (Hypothesis 2). The mediating effect of negative emotions in the link between negative life events and procrastination will also be moderated by rumination, with this indirect effect being stronger for adolescents with a higher rumination (Hypothesis 3). 

### 1.4. Gender Difference

In addition, gender differences should also be considered. First, adolescence is a time when sex differences develop rapidly, and the establishment of specific gender role is an important social development task for adolescents—namely, they have to acquire appropriate gender behavior patterns and values [46,47]. Following this, previous studies have found that there exist significant gender differences regarding negative life events, negative emotions, rumination and procrastination [2,25,47]. Specifically, females usually report slightly more stressful life events and perceive more negative emotions including depressive symptoms, anxiety symptoms and stress symptoms than males, and this difference is particularly significant in adolescents [48,49]. At the same time, prior studies also have suggested that females ruminate more than males, and adolescent girls are more likely to be engaged into rumination and suffer from it [47]. Rumination is usually considered an effective way to explain the gender differences regarding negative emotions [49]. Specifically, one reason why females experience more negative emotions is that they ruminate more after experiencing adverse life events than men do. Regarding procrastination, it is prominent among young adolescents on account of their inadequate regulation ability [3], and significant gender differences also have been found in procrastination and its relationships with other variables [50]. For example, studies found that personality traits play different roles in procrastination in different gender groups [25], and adolescent girls reported more procrastination experiences [51]. Therefore, gender differences should be taken into account when examining the potential mechanisms and conditions underlying the relationships between negative life events and procrastination. However, even though previous studies have been carefully combed, a precise and powerful hypothesis about the gender differences of the proposed moderated mediation model in this study still cannot be put forward. Therefore, gender differences in the proposed moderated mediation model will be considered as an exploratory hypothesis. Gender differences may exist in the proposed moderated mediation model (Hypothesis 4).

### 1.5. The Present Study

**Figure 1:**
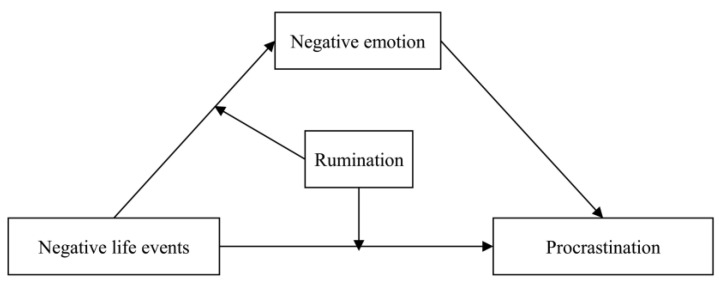
Conceptual model.

Methodology research has shown that the moderated mediation model could reveal more insightful knowledge for us in understanding the potential mechanisms and conditions underlying the link between the two variables [52]. This study investigated the mediating and moderating mechanisms underlying the link between negative life events and procrastination by testing a moderated mediation model (see Figure 1). Negative emotions were treated as a mediator to explain how negative life events were linked to procrastination. Rumination was considered as a moderator to answer when the effects of negative life events on procrastination were more significant. In addition, potential gender differences in this proposed moderated mediation model have also been taken into account. 

## 2. Materials and Methods

### 2.1. Participants

A total of 780 middle school students (52.2% females) were recruited. All of the participants were between 11 and 15 years old, with a mean age of 12.92 years old (*SD* = 0.902). Three hundred and fifty-one (45.0%) of them were Grade 7 students; two hundred and eighty-nine (37.1%) of them were Grade 8 students; and one hundred and forty (17.9%) of them were Grade 9 students. 

### 2.2. Procedure

The ethical committee at the correspondence author’s unit approved this study before the formal investigation, and a signed consent form was collected from each student’s parents. Besides this, informed consent was also obtained from the schools, teachers, and participants before this survey. A paper-and-pencil survey was adopted to collect information about negative life events, procrastination, negative emotions, rumination and demographic variables. All of the participants completed the survey in 30 min in their classroom after being informed of the requirements and the confidentiality of this survey.

### 2.3. Measurements

#### 2.3.1. Negative Life Events

The Chinese version of the adolescent self-rating life events checklist (ASLEC) was adopted to assess negative life events [14]. This scale has been used in a sample of Chinese middle school students with good reliability and validity [14]. The participants were required to evaluate whether or not they had experienced the life events described in each of the 27 items. If they answered “yes” (e.g., “Stay away from family for a long time and cannot be reunited”), they were requested to assess the influence of the life events on a Likert-type 5-point scale. Cronbach’s α was 0.909.

#### 2.3.2. Procrastination

The Chinese version of the irrational procrastination scale was used to assess the frequency of procrastination [1]. Participants were invited to respond to the 9 items on a Likert-type 5-point scale (e.g., “My life would be better if I did some activities or tasks earlier”). The CFA indexes generated by Amos 21.0 showed that this one-dimensional measurement model had a good fit with the data collected from Chinese middle school students: *χ*^2^*/df* = 3.60, RMSEA = 0.06, CFI = 0.99, NFI = 0.97, GFI = 0.99. Cronbach’s α was 0.713.

#### 2.3.3. Negative Emotions

The degree of participants’ subjective negative emotions was assessed using the Chinese version of depression-anxiety-stress scale (DASS), which has shown a good reliability and validity in a sample of Chinese middle students [53]. Participants were invited to respond to the 21 items on a 4-point Likert-type scale. Sample items are “I felt that life was meaningless”, “I felt scared without any good reason”, and “I found it difficult to relax”. Cronbach’s alpha was 0.918.

#### 2.3.4. Rumination

The Chinese short version of ruminative response scale was adopted to assess the degree of participants’ rumination [54]. This scale has shown good reliability and validity in a sample of Chinese middle school students [54]. Participants responded to the 10 items on a 4-point Likert-type scale (e.g., “Write down what you are thinking and analyze it” and “Go someplace alone to think about your feelings”). Cronbach’s α = 0.821. 

### 2.4. Statistical Analyses

SPSS 23.0 was adopted to sort and analyze the research data. Firstly, descriptive statistics and Pearson correlations were conducted for the main variables. Secondly, the SPSS macro PROCESS was adopted to test the hypothesized model, which was developed and widely used to test complex models with mediating and moderating roles [55,56], and all the analyses were conducted using a 5000-bootstraps sample to generate 95% bias-corrected confidence intervals (CI) for all the indexes; if zero is not included in the 95% CI, the effects were regarded as significant. In particular, model 4 was adopted to examine the mediating effect of negative emotions, and the model 8 was then employed to test the hypothesized moderated mediating model with negative emotions in a mediating role and rumination in the moderating role. Thirdly, the potential interaction effects were illustrated by simple slopes analyses. Moreover, the potential gender differences in the proposed moderated mediation model were also examined by testing the proposed model against samples of males and females. 

## 3. Results

### 3.1. Descriptive Statistics and Correlation Analyses

Descriptive analysis results were presented in Table 1. As hypothesized, all of these observed variables were positively correlated with each other, and they showed no significant correlation with age.

### 3.2. Testing for the Proposed Moderated Mediation Model

The SPSS macro PROCESS (Model 4) was employed to test the mediation model, firstly in the samples of males and females. The main results were presented in Table 2.

After controlling for grade (which is closely correlated with social experience), negative life events were positively associated with procrastination for both males and females. Negative life events were also positively associated with negative emotions for both males and females. Negative emotions were also positively associated with procrastination for both males and females. Besides this, the 95% confidence interval of total effect, direct effect, and indirect effect for females did not contain zero. The indirect effect accounted for 59.22% of the total effect in female samples. The 95% confidence interval of total effect and indirect effect for males did not contain zero, whereas the 95% confidence interval of direct effect for males contained zero. The indirect effect accounted for 62.13% of the total effect in male samples.

In addition, with the aim to examine the potential gender differences, the SPSS macro PROCESS (Model 8) was employed to examine the moderated mediation model in male and female participants, respectively. The main results were presented in Table 3.

After controlling for grade, negative life events were also positively associated with negative emotions for both males and females. Negative emotions were also positively associated with procrastination for both males and females, whereas the link between negative life events and procrastination was not significant for neither males or females. Furthermore, Sobel tests found that the mediating effect of negative emotions was significant for both males (*z* = 2.471, *p* < 0.05) and females (*z* = 5.100, *p* < 0.001).

Additionally, a significant negative life events × rumination interaction effect on negative emotions in the model of the mediator variable for males was found, whereas it could not be found in the model of the mediator variable for females. There were significant negative life events × rumination interaction effects in the model of dependent variable for both males and females. These results indicated that the effects of negative life events on procrastination and negative emotions were all moderated by rumination for males, whereas only the effect of negative life events on procrastination could be moderated by rumination for females.

**Figure 2:**
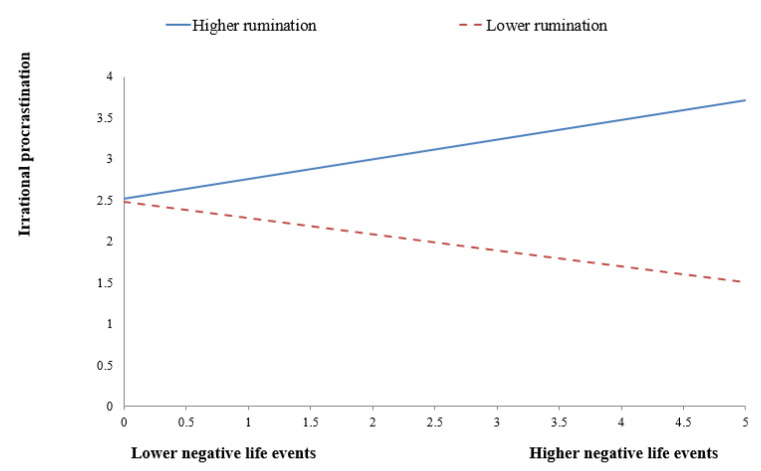
Rumination moderates the regression coefficient of negative life events on negative emotions in the sample of males.

**Figure 3:**
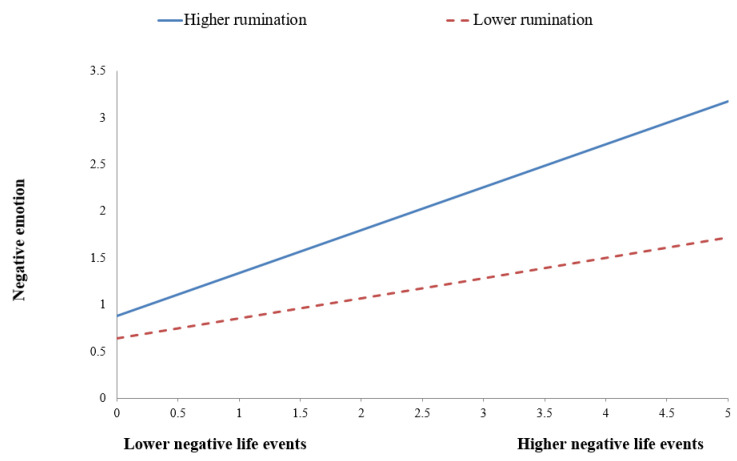
Rumination moderates the regression coefficient of negative life events on procrastination in the sample of males.

**Figure 4:**
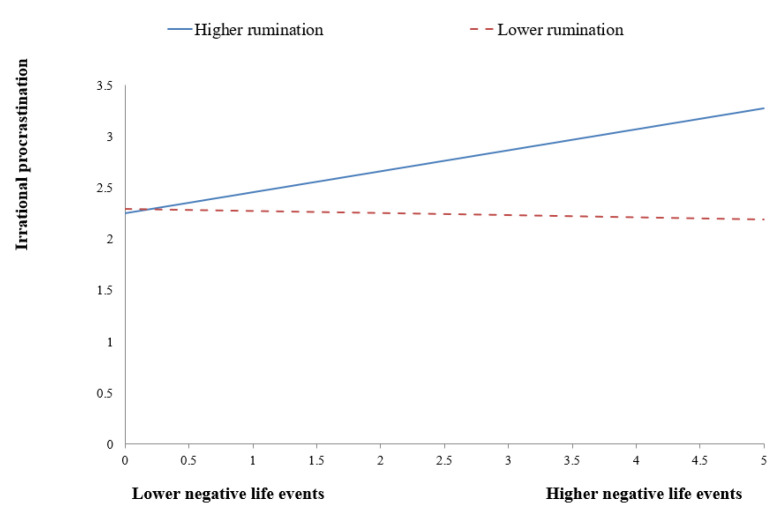
Rumination moderates the regression coefficient of negative life events on procrastination in the sample of females.

To decompose these interactions more clearly, simple slope analyses were also conducted. As shown in Figure 2, for males, the effect of negative life events on negative emotions was stronger for the high-rumination group (*B* = 0.459, *t =* 7.847, *p* < 0.001) than for the low-rumination group (*B* = 0.217, *t =* 2.852, *p* < 0.01). As shown in Figure 3, for males, the effect of negative life events on procrastination was significant and positive for the high-rumination group (*B* = 0.239, *t =* 3.070, *p* < 0.01), whereas it was significant and negative for the low-rumination group (*B* = −0.194, *t =* −2.357, *p <* 0.05). As shown in Figure 4, for females, the effect of negative life events on procrastination was significant for the high-rumination group (*B* = 0.204, *t =* 3.157, *p* < 0.01), whereas it was not significant for the low-rumination group (*B* = −0.020, *t =* −0.243, *p* > 0.05).

Furthermore, the conditional direct effect analyses also indicated that for males the conditional direct effect among the high-rumination group was positively and significantly different from zero, whereas it was negatively and significantly different from zero among the low-rumination group. For females, only the conditional direct effect among the high-rumination group was positively and significantly different from zero. Namely, the direct effect of negative life events on procrastination for females with higher rumination was positive and significant. The direct effect of negative life events on procrastination was positive for males with higher rumination, whereas it was negative for males with lower rumination. The conditional indirect effect analyses indicated that for males, all of the conditional indirect effects were positively and significantly different from zero. Specifically, the indirect effect of negative life events on procrastination through negative emotions was stronger for males with higher rumination.

## 4. Discussion

Although prior studies have shed light on the influencing factors of procrastination, such as workload [57], low self-esteem [19] and personality traits [25], none have considered the role of negative life events in procrastination and how and when negative life events are linked to procrastination. To enrich our knowledge, this study took negative emotions as a mediator and rumination as a moderator and proposed a moderated mediation model. In addition, gender may also play a role in the mechanism underlying the link between negative life events and procrastination. Therefore, gender differences were taken into account in the proposed moderated mediation model. 

The results indicated that negative life events were positively associated with procrastination for both males and females; thus, Hypothesis 1a was supported. Negative emotions mediated the relation between negative life events and procrastination for both males and females; thus Hypothesis 1b, was supported. Moreover, gender differences were found in this mediation model. Specifically, the link between negative life events and procrastination was totally mediated by negative emotions for females, whereas this process was partly mediated by negative emotions for males. Moreover, rumination played an important role as a moderator, and this moderating effect showed gender differences. Both the direct and indirect effect of negative life events on procrastination could be moderated by rumination for males, whereas only the direct effect of negative life events on procrastination could be moderated by rumination for females. Specifically, negative life events had a negative direct effect on procrastination for males with low rumination, whereas this direct effect was positive for males with high rumination. In addition, negative life events exerted a positive and significant effect on procrastination only for females with higher rumination. Moreover, the indirect effect of negative emotions was stronger for males with higher rumination. Therefore, Hypothesis 2, Hypothesis 3 and Hypothesis 4 were supported. These results provide new perspectives for us to interpret the bridge (mediation) and contextual (moderation and gender differences) factors in the link between negative life events and procrastination. In other words, these findings may enrich our knowledge of how negative life events influence procrastination, and for whom this effect is more significant.

### 4.1. Negative Life Events and Procrastination

Our results showed that negative life events were positively associated with procrastination among adolescents. Previous studies have shown that helplessness caused by negative life events may damage adolescents’ sense of self-worth and self-efficacy [18,58], making them doubt their ability to do what they can do well [3,8]. If they continue in these irrational beliefs, they may protect their self-esteem by self-handicapping and putting off what they should do as late as possible [21]. This behavioral habit may push them into the vicious circle of self-limitation and procrastination. Moreover, given that negative life events can consume an individual’s self-control resources, negative life events may naturally result in low self-control [59]. According to self-regulation failure theory [4], procrastination as a manifestation of self-regulatory failure may result from adolescents’ low self-control caused by negative life events. 

### 4.2. Negative Emotions as a Mediator

Our study also showed that negative emotions could mediate the link between negative life events and procrastination. Negative life events, as environmental factors, can affect individuals’ behavior by inducing their negative emotions. Previous studies have suggested that adolescents suffering from more negative life events tend to perceive more stress, depression and anxiety symptoms [60]. Given that negative emotions are the causes of irrational delay, individuals suffering from negative emotions, including stress, depression and anxiety symptoms, are likely to irrationally put off their duties [3]. Thus, adolescents suffering from more negative life events may be drawn into negative emotions, which may exhaust their time or energy to do what they should do and in turn lead to repeated procrastination of these tasks. Moreover, gender differences were also found in this mediating effect. Specifically, negative emotions could totally mediate the link between negative life events and males’ procrastination, whereas it played a partially mediating effect on this relation among females. According to social role theory, gender differences could be explained by the social expectations for both males and females [61]. More specifically, males are expected to be active and rational in solving the problems they face, whereas females are allowed to be more passive and emotional. Hence, males are more likely to do anything to solve the problems they face when suffering from negative life events. However, females may be more dependent and involved in irrational behaviors (such as procrastination) to cope with the negative life events. Therefore, negative life events were positively associated with the likelihood of females’ procrastination. 

### 4.3. Rumination as a Moderator

Our study illustrates that rumination played an important role as a moderator. Negative life events exerted a direct and positive effect on procrastination for both males and females with higher rumination. The results indicated that rumination could exacerbate the potential adverse effects of environmental risk factors (e.g., negative life events) on adolescents’ behavior adaptation (e.g., procrastination). Previous studies have indicated that rumination aggravates emotional or behavioral adaptation problems by activating negative associative memory networks, interfering with attention and instrumental behavior, impairing problem solving and impacting social support networks [62]. Consequently, when suffering from negative life events, adolescents with high rumination may be overwhelmed by the activated negative associative memory networks and have poor self-efficacy and self-regulatory capacity. Thus, they tend to develop emotional and behavioral problems. According to a meta-analysis study conducted by Steel [4], the nature of procrastination is self-regulatory failure. Adolescents with high rumination may be tired of coping with negative life events and have no time and energy to achieve self-regulation and complete planned tasks. Therefore, adolescents with high rumination may be involved in more procrastination when suffering from negative life events. 

### 4.4. Gender Difference

In addition, gender differences were also found in this study. First, the moderating direct effect showed gender differences. Specifically, the direct effect was negative and significant for males with lower rumination, whereas it was not significant for females with lower rumination. These results fit well into the social role theory [61], which indicates that social standards introduce different role expectations for males and females. Males are expected to solve the problems they face rationally and carry out planned tasks smoothly in every possible response; thus, males with lower rumination were more likely to transform stress induced by negative life events into motivation and do what they should do on schedule. Second, the moderating indirect effect of rumination also showed gender differences. For males, the indirect effect could be moderated by rumination, whereas this moderating effect could not be found in females. People usually have more acceptance and tolerance for females’ negative emotions, and males are required to hide their negative emotions and solve the problems they face rationally, especially in Chinese culture [61]. This may weaken the moderating effect of rumination in adolescent girls. On the other hand, society has low tolerance for males who disclose their negative emotions, and they thus usually tend to suppress their negative emotions. In these circumstances, males with higher rumination may think over the causes and potential adverse consequences of negative life events repeatedly, have no time or energy to explore ways to cope with negative life events and therefore suffer from more negative emotions. Given that more negative emotions are related to more procrastination [4,62], the mediating effect of negative emotions was more significant for males with higher rumination than for those with lower rumination. 

## 5. Limitations and Implications

Several limitations of this research should be stated. First, the causal directions among negative life events, negative emotions and procrastination cannot be inferred from this study. Longitudinal research can monitor changes in the levels of negative emotions and procrastination among individuals suffering from negative life events with different levels of rumination. Future studies need to adopt longitudinal research to strictly confirm both the causal relationships between negative life events and procrastination and the causal relationships between negative emotions and procrastination. Second, since all participants in this study were recruited from two middle schools in China, the external validity of this study was limited. It is necessary for future research to take other participants from different cultural backgrounds into consideration. Besides this, social desirability and other biases restricted the validity of the data obtained by the self-report method; thus, a method for multidimensional scaling needs to be adopted in future studies to collect more objective data. Thirdly, the gender differences need further direct examination, so as to clearly address this issue.

Adolescence is a critical period in which individuals need to complete the transition from childhood to adulthood. Confronted with academic pressure and other potential growth crises, adolescents are likely to experience multiple negative life events [63], which increase the risk of being involved in emotional and behavioral adaptation problems. This study highlights the effects of negative emotions and rumination to enrich our knowledge about the impact of negative life events on adolescents’ procrastination. Our findings could help us to understand the relationship between negative life events and adolescents’ behavioral problems and the mechanism and individual differences underlying this link. Besides this, practical implications for both parents and educators could be inferred from this study. First, a previous study showed that a positive coping style could moderate the adverse effect of negative life events on adolescents’ behavioral adaptation [64]. Therefore, educators or parents can offer constructive suggestions for adolescents to develop positive coping styles and cope with negative life events more actively and effectively. Second, given that negative emotions could mediate the link between negative life events and procrastination, educators or parents should help adolescents suffering from negative life events to vent their negative emotions reasonably and in a timely manner and motivate them to devote more energy and cognitive resources to dealing with their main tasks. In addition, individual differences should also be taken into consideration to reduce adolescents’ procrastination. For adolescents with higher rumination, they should focus on dealing with the potential negative emotions caused by negative life events. A previous study demonstrated that individuals could reduce their rumination through multiple kinds of mindfulness training [65]. Therefore, adolescents with higher rumination could reduce their procrastination by coping with negative life events and negative emotions scientifically as well as by training their mindfulness skills. Moreover, considering the gender differences in the underlying mechanisms and conditions of the link between influencing factors and adolescents’ procrastination, educators or parents should take gender into account when helping adolescents overcome procrastination—in particular, adolescent girls should be paid more attention to, as a consequence of their susceptibility to negative emotions and procrastination, and more targeted measures should be taken to relieve their negative events and negative emotions. 

## Figures and Tables

**Table 1:** Descriptive statistics and interrelations among all of the observed variables and age.

Variables	*M (SD)*	1	2	3	4	5
1. Negative life events	1.176(0.735)	1				
2. Negative emotion	0.767(0.543)	0.526 **	1			
3. Procrastination	2.687(0.673)	0.245 **	0.328 **	1		
4. Rumination	2.224(0.591)	0.292 **	0.284 **	0.076 *	1	
5. Age	12.92(0.902)	−0.006	−0.038	0.020	0.021	1

** *p* < 0.01, * *p* < 0.05.

**Table 2:** Regression results for the mediation model.

*Model*	Results for the Sample of Males (n = 373)										Results for the Sample of Females (n = 407)									
***Model 1: Total effect model***																				
	***R***	***R* ^2^**		***F***		***df*_1_**		***df*_2_**		***p***	***R***	***R* ^2^**		***F***		***df*_1_**		***df*_2_**		***p***
	0.188	0.036		5.971		2		370		<0.01	0.296	0.087		14.384		2		404		<0.001
	***B***		***SE***		***t***				***p***		***B***	***SE***		***t***				***p***		
Constant	2.531		0.106		23.794 ***				<0.001		2.332	0.089		26.291 ***				<0.001		
Grade	−0.006		0.047		−0.119				>0.05		−0.002	0.041		0.039				>0.05		
NLEs	0.176		0.051		3.435 ***				<0.001		0.265	0.050		5.266 ***				<0.001		
***Model 2: Mediator variable model***																				
	***R***	***R* ^2^**		***F***		***df*_1_**		***df*_2_**		***p***	***R***	***R* ^2^**		***F***		***df*_1_**		***df*_2_**		***p***
	0.535	0.286		56.380		2		370		<0.001	0.527	0.278		60.796		2		404		<0.001
	***B***		***SE***		***t***				***p***		***B***	***SE***		***t***				***p***		
Constant	0.317		0.075		4.252 ***				<0.001		0.373	0.064		5.801 ***				<0.001		
Grade	−0.007		0.031		−0.224				>0.05		−0.039	0.031		−1.252				>0.05		
NLEs	0.424		0.041		10.445 ***				<0.001		0.365	0.033		11.008 ***				<0.001		
***Model 3: Dependent variable model***																				
	***R***	***R* ^2^**		***F***		***df*_1_**		***df*_2_**		***p***	***R***	***R* ^2^**	***F***		***df*_1_**		***df*_2_**			***p***
	0.264	0.070		8.221		3		369		<0.001	0.408	0.167	24.218		3		403			<0.001
	***B***		***SE***		***t***				***p***		***B***	***SE***		***t***				***p***		
Constant	2.450		0.105		23.388 ***				<0.001		2.172	0.083		26.052 ***				<0.001		
Grade	−0.004		0.046		−0.083				>0.05		0.018	0.039		0.470				>0.05		
NLEs	0.067		0.059		1.124				>0.05		0.108	0.053		2.031 *				<0.05		
NE	0.257		0.073		3.514 ***				<0.001		0.430	0.072		5.955 ***				<0.001		
***Specific effect analysis***																				
	***B***		**SE**		**LLCI**		**ULCI**		**Ratio**		***B***		**SE**		**LLCI**		**ULCI**		**Ratio**	
TE	0.176		0.051		0.078		0.276				0.265		0.050		0.166		0.364			
DE	0.066		0.059		−0.050		0.183				0.108		0.053		0.004		0.213		40.78%	
IE	0.109		0.032		0.047		0.175		62.13%		0.157		0.028		0.104		0.215		59.22%	

Unstandardized regression coefficients are reported. Bootstrap sample size = 5000. NELs = Negative life events, NE = Negative emotion, TE = Total effect, DE = Direct effect, IE = Indirect effect, LL = low limit, CI = confidence interval, UL = upper limit. Ratio = Ratio of direct effect or indirect effect to total effect, * *p* < 0.05,. *** *p* < 0.001.

**Table 3:** Regression results for the conditional indirect effects (moderated mediation).

*Model*	Results for the Sample of Males (n = 373)									Results for the Sample of Females (n = 407)											
***Model 1: Mediator variable model***																					
	***R***	***R* ^2^**		***F***		***df*_1_**	***df*_2_**		***p***	***R***	***R* ^2^**			***F***			***df*_1_**		***df*_2_**		***p***
	0.585	0.342		31.644		4	368		<0.001	0.537	0.289			32.282			4		402		<0.001
	***B***		***SE***		***t***			***p***		***B***		***SE***			***t***					***p***	
Constant	0.758		0.057		13.229 ***			<0.001		0.826		0.059			13.909 ***					<0.001	
Grade	0.005		0.029		0.179			>0.05		−0.051		0.031			−1.649					>0.05	
NLEs	0.338		0.041		8.277 ***			<0.001		0.341		0.035			9.815 ***					<0.001	
Rum	0.206		0.047		4.344 ***			<0.001		0.095		0.043			2.219 *					<0.05	
NLEs × Rum	0.205		0.091		2.250 *			<0.05		0.040		0.050			0.432					>0.05	
***Model 2: Dependent variable model***																					
	***R***	***R* ^2^**		***F***		***df*_1_**	***df*_2_**		***p***	***R***	***R* ^2^**		***F***			***df*_1_**		***df*_2_**			***p***
	0.345	0.118		8.403		5	367		<0.001	0.430	0.185		15.416			5		401			<0.001
	***B***		***SE***		***t***			***p***		***B***		***SE***			***t***					***p***	
Constant	2.494		0.010		25.041 ***			<0.001		2.271		0.092			24.718 ***					<0.001	
Grade	0.012		0.045		0.274			>0.05		0.021		0.039			0.544					>0.05	
NLEs	0.023		0.053		0.424			>0.05		0.092		0.054			1.699					>0.05	
NE	0.202		0.078		2.581 *			<0.05		0.431		0.072			5.999 ***					<0.001	
Rum	0.030		0.067		0.444			>0.05		−0.037		0.063			−0.591					>0.05	
NLEs × Rum	0.367		0.102		3.599 ***			<0.001		0.190		0.086			2.212 *					<0.05	
***Conditional direct effect analysis at values of rumination (M ± SD)***																					
	***B***		**SE**		**LLCI**			**ULCI**		***B***		**SE**			**LLCI**					**ULCI**	
M − 1SD	−0.189		0.082		−0.349			−0.028		−0.023		0.084			−0.188					0.142	
M	0.023		0.053		−0.082			0.128		0.092		0.054			−0.014					0.199	
M + 1SD	0.234		0.077		0.083			0.385		0.207		0.065			0.079					0.335	
***Conditional indirect effect analysis at values of rumination (M ± SD)***																					
	***B***		**SE**		**LLCI**			**ULCI**		***B***		**SE**			**LLCI**					**ULCI**	
M − 1SD	0.045		0.021		0.013			0.095		0.137		0.030			0.087					0.204	
M	0.068		0.027		0.018			0.123		0.147		0.026			0.099					0.205	
M + 1SD	0.092		0.038		0.022			0.170		0.157		0.029			0.105					0.221	

Unstandardized regression coefficients are reported. Bootstrap sample size = 5000. NELs = Negative life events, NE = Negative emotion, Rum = Rumination, LL = low limit, CI = confidence interval, UL = upper limit. * *p* < 0.05, *** *p* < 0.001.
